# Chronological and biological age stratify survival after robot-assisted radical cystectomy for bladder cancer: a pragmatic age-ECOG risk score

**DOI:** 10.1007/s00345-026-06294-4

**Published:** 2026-02-17

**Authors:** Jakob Kohler, Leonhard Buck, Konrad Hügelmann, Reha-Baris Incesu, Hans Christoph von Knobloch, Tim Krumm, Julian Risch, Patricia Schließer, Jakob Christoph Voran, Oscar Weische, Marie Weiss, Jonas Jarczyk, Philipp Nuhn, Severin Rodler

**Affiliations:** 1 Department of Urology, University Hospital Schleswig-Holstein (UKSH), Arnold-Heller-Strasse 1-3, Campus Kiel, 24105 Kiel, Germany; 2 Kurt-Semm Center for laparoscopic and robotic surgery, University Hospital Schleswig- Holstein Campus Kiel, Kiel, Germany; 3 Department of Internal Medicine III, Cardiology and Angiology, University Hospital Schleswig-Holstein, Campus Kiel, Kiel, Germany

**Keywords:** Urothelial carcinoma, Radical cystectomy, Robotic surgery, Risk stratification, ECOG performance status, Enhanced recovery after surgery

## Abstract

**Purpose:**

Chronological age influences selection for radical cystectomy in bladder cancer, yet biological vulnerability may be more informative. We assessed perioperative outcomes and overall survival after robot-assisted radical cystectomy (RARC) and developed a pragmatic age–Eastern Cooperative Oncology Group (ECOG) risk score.

**Methods:**

Consecutive RARC patients (August 2013–December 2024) were analyzed retrospectively and grouped by age (< 75, 75–79, 80–84, ≥ 85 years). Ninety-day complications were graded by Clavien–Dindo classification. Overall survival (OS) was estimated by Kaplan–Meier methods. An age threshold for overall mortality was derived by receiver operating characteristic analysis (Youden index). The risk score assigned one point each for age above the cut-off and ECOG performance status ≥ 2. Cox regression adjusted for Charlson Comorbidity Index and pathological T stage.

**Results:**

Among 171 patients, length of stay and overall complications did not differ statistically across age strata, although comparisons are limited by selection and the small ≥ 85 subgroup. OS differed by age cohort (*p* = 0.00013) with an optimal age threshold of 76.5 years (*p* < 0.0001). ECOG ≥ 2 predicted worse survival (*p* < 0.0001), and the score separated 1, 2 and 3 score groups (*p* < 0.0001). On multivariable analysis, age > 76.5 years remained independently associated with worse overall survival (hazard ratio 2.72, 95% confidence interval 1.58–4.68; *p* < 0.001).

**Conclusion:**

RARC is feasible across advanced age strata in selected patients, but survival varies substantially. A simple age threshold plus ECOG performance status may aid counseling and perioperative planning and warrants external validation.

**Supplementary Information:**

The online version contains supplementary material available at 10.1007/s00345-026-06294-4.

## Introduction

Bladder cancer is predominantly a disease of older adults, and a substantial proportion of patients present with non-metastatic muscle-invasive disease requiring curative-intent treatment [1, 2]. For eligible patients, radical cystectomy with pelvic lymph node dissection within a multidisciplinary pathway remains a cornerstone of management for non-metastatic muscle-invasive bladder cancer [2]. However, older patients are less likely than younger patients to undergo radical cystectomy, largely reflecting concerns regarding perioperative morbidity and early mortality [1].

Contemporary outcome data confirm that these concerns are not trivial, particularly at very advanced age [1]. In a recent systematic review and meta-analysis, ninety-day mortality after radical cystectomy was approximately 11% in cohorts aged 80 years or older and 7% in cohorts aged 75 years or older, with significantly higher odds of ninety-day mortality in patients aged 80 years or older compared with younger counterparts [1]. Within enhanced recovery after surgery (ERAS) pathways, intensive care unit admission after radical cystectomy appears uncommon, yet unplanned transfer is associated with higher age and comorbidity burden and is linked to prolonged length of stay [3]. At the same time, chronological age alone is an imprecise proxy for biological vulnerability, and functional reserve is increasingly recognized as a key determinant of perioperative risk and longer-term outcomes [1, 4, 5]. Frailty is prevalent among patients undergoing radical cystectomy and is consistently associated with adverse perioperative outcomes across multiple frailty definitions and study designs [4, 5].

Robot-assisted radical cystectomy (RARC) has gained broad adoption, and randomized data demonstrate non-inferior oncologic outcomes compared with open radical cystectomy [6]. Nevertheless, evidence supporting pragmatic, readily available preoperative stratification tools tailored to RARC, especially in very elderly patients, remains limited [1, 6]. Eastern Cooperative Oncology Group (ECOG) performance status is routinely documented and, when combined with chronological age, has been shown to stratify survival after radical nephroureterectomy for upper-tract urothelial carcinoma using a simple risk score framework [7].

Building on our prior institutional work demonstrating perioperative non-inferiority of RARC in overweight subgroups, we sought to interrogate age- and performance-status–related heterogeneity specifically within the RARC cohort [8].

Accordingly, the aim of the present study was to evaluate the association of chronological age and ECOG performance status with overall survival after RARC and to derive a pragmatic combined risk score to support counseling and perioperative planning [7, 8].

## Materials and methods

### Study design, setting, and ethics

This retrospective single-center cohort study was conducted at the Department of Urology, University Hospital Schleswig-Holstein (UKSH), Kiel, Germany. The study was approved by the Ethics Committee of the Medical Faculty, Christian-Albrechts-University of Kiel (D 402/25) and conducted in accordance with the Declaration of Helsinki. For vital status ascertainment, linkage to official mortality records via the statistical offices of Hamburg and Schleswig-Holstein was additionally approved (D 515/25).

### Patient cohort and eligibility

All consecutive patients undergoing radical cystectomy with urinary diversion for bladder cancer between August 2013 and December 2024 were screened. The present analysis was restricted to patients treated with RARC. Patients with non-urothelial histologies or non-malignant indications were excluded per the institutional cystectomy database definition. Patients were stratified into four prespecified age cohorts: <75, 75–79, 80–84, and ≥ 85 years.

### Surgical approach and perioperative management

RARC was performed using the da Vinci surgical system (Si and Xi platforms). Pelvic lymph node dissection followed a standard template. All diversion types were included (ureterocutaneostomy, ileal conduit, orthotopic neobladder, and continent pouch), selected based on patient factors, tumor stage, and surgeon preference.

### Variables and endpoint definitions

Baseline variables were age, sex, American Society of Anesthesiologists (ASA) physical status, and ECOG performance status. Perioperative outcomes included length of stay (operation to discharge, days) and 90-day postoperative complications graded by Clavien–Dindo; the highest grade per patient was used. Overall survival was defined from surgery to death from any cause or last follow-up. Deaths were ascertained via official records; patients were censored at last known alive, with administrative censoring on 28 December 2025.

### Risk stratification (chronological + biological age)

ECOG performance status was dichotomized (0–1 vs. ≥ 2). An age threshold for overall mortality (death at any time during follow-up vs. censored) was derived by receiver operating characteristics analysis using the Youden index, yielding a cut-off of 76.5 years. A combined age–ECOG risk score was derived by assigning one point each for age > 76.5 years and ECOG ≥ 2 and categorizing patients into three levels (score 1–3): score 1 (0 risk factors), score 2 (1 risk factor), and score 3 (2 risk factors).

### Statistical analysis

Continuous variables are reported as median (range) and categorical variables as frequency (percentage). Comparisons across age cohorts used Kruskal–Wallis tests (continuous) and χ² or Fisher’s exact tests (categorical). Overall survival was analyzed by Kaplan–Meier methods with log-rank testing; Breslow testing was additionally applied where appropriate. Multivariable Cox regression for overall survival included age (> 76.5 vs. ≤ 76.5), ECOG (≥ 2 vs. 0–1), ASA score, Charlson Comorbidity Index, pathological T stage (reference ≤pT1), pathological nodal status (N + vs. N0), and sex, using complete-case analysis. Proportional hazards were assessed with Schoenfeld residuals; minor deviations were noted but visual inspection did not suggest clinically relevant violations. All tests were two-sided (*p* < 0.05). Analyses were performed using R version 4.5.1.

## Results

### Patient cohort and baseline characteristics

Overall, 171 patients undergoing radical cystectomy were included and stratified into four age cohorts: <75 years (*n* = 98), 75–79 years (*n* = 35), 80–84 years (*n* = 32), and ≥ 85 years (*n* = 6). Median age was 65, 77, 82, and 86 years across cohorts, respectively (*p* < 0.001, Table 1). Sex distribution did not differ significantly between groups (female: 5.1%, 2.9%, 12.5%, and 0%; *p* = 0.307). Comorbidity burden and functional status increased with age: there was a trend toward higher ASA scores in older cohorts (*p* = 0.084), and ECOG performance status differed significantly, with fewer patients presenting with ECOG performance status 0 in older cohorts (*p* < 0.001, Table 1).

### Perioperative outcomes

Median time from operation to discharge was 15, 17, 15, and 12 days across cohorts 1–4, respectively, without statistically significant differences (*p* = 0.270, *Online Resource 1*). Perioperative complications occurred in 55.1%, 62.9%, 50.0%, and 66.7% of patients, respectively (*p* = 0.695). Among patients experiencing complications, the distribution of Clavien–Dindo grades did not differ significantly across age cohorts (*p* = 0.196, *Online Resource 1*). Given the small ≥ 85-year subgroup and non-random selection for surgery, these findings should be interpreted as descriptive and should not be taken to demonstrate perioperative equivalence across age strata. We focused on overall complication burden and highest-grade events per patient within 90 days; more granular benchmarking constructs (e.g., textbook outcome composites) were not evaluated in this retrospective dataset.

### Overall survival and risk stratification

**Fig. 1:**
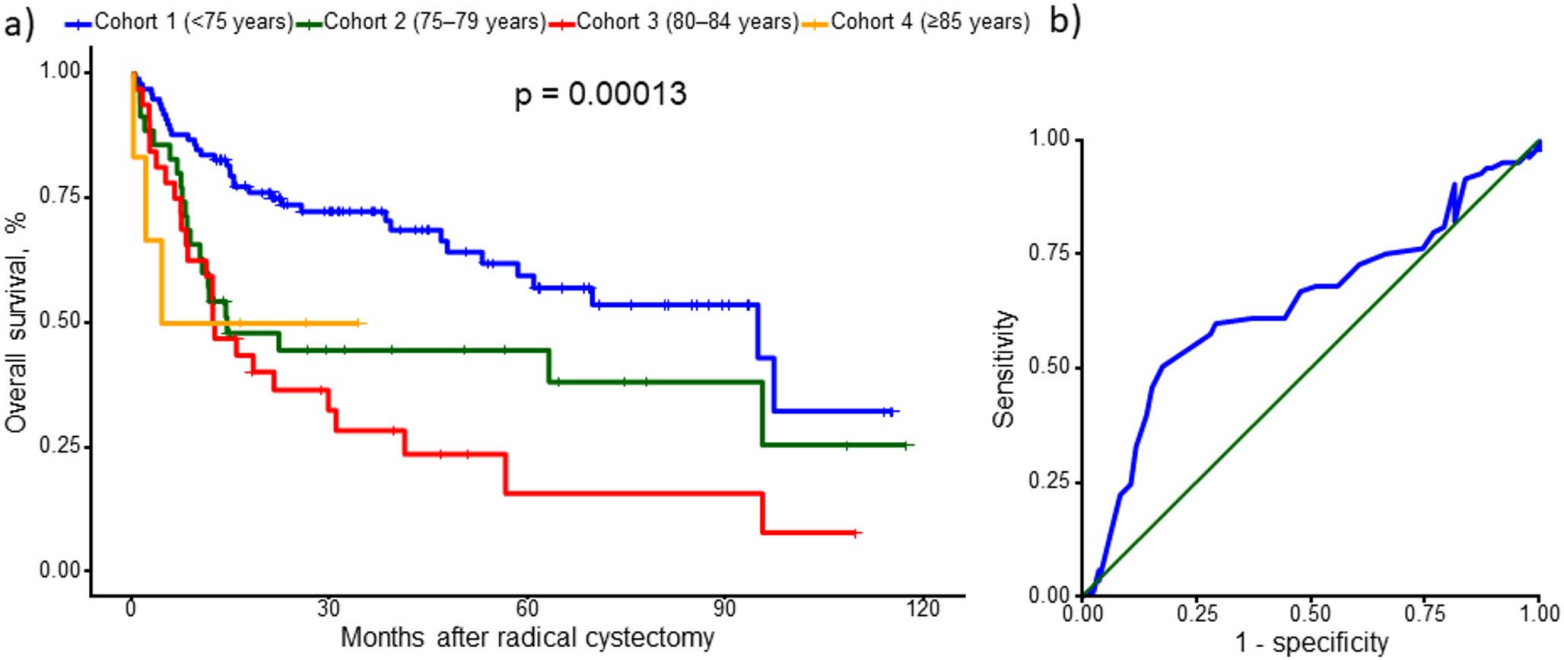
Overall survival after radical cystectomy by age cohort and discrimination of the survival model. (a) Kaplan–Meier estimates of overall survival stratified by age (< 75, 75–79, 80–84, and ≥ 85 years); survival curves were compared using the log-rank test (*p* = 0.00013). Tick marks indicate censored observations (b) Receiver operating characteristic curve evaluating model discrimination; the diagonal line indicates the no-discrimination reference

**Fig. 2:**
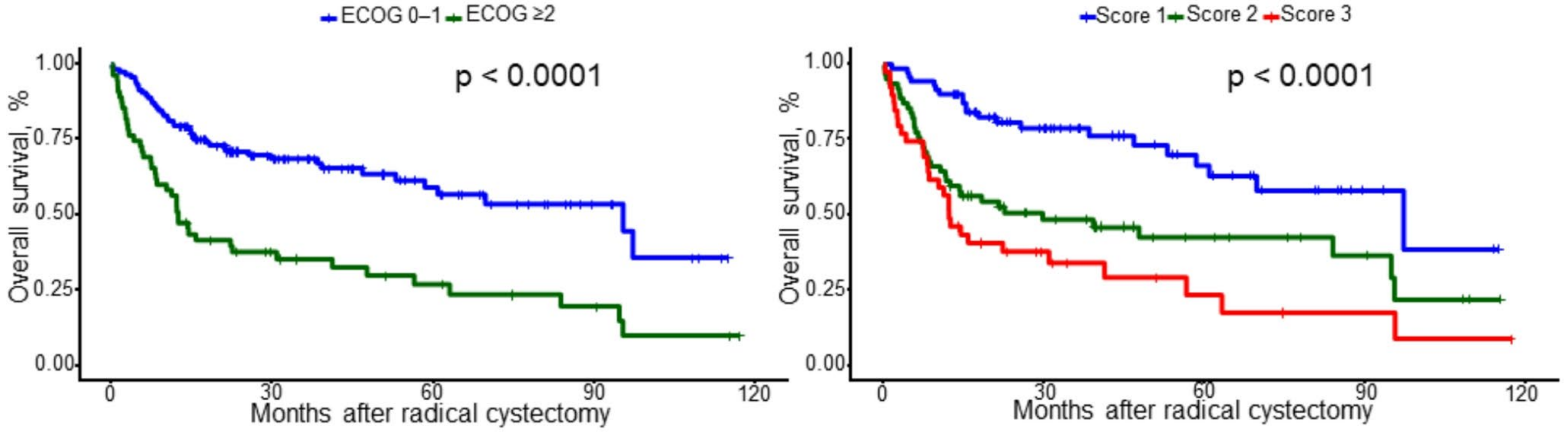
Overall survival after radical cystectomy according to functional status and composite risk stratification. (a) Kaplan–Meier estimates of overall survival stratified by Eastern Cooperative Oncology Group performance status (ECOG 0–1 vs. ECOG ≥ 2); curves were compared using the log-rank test (*p* < 0.0001). Tick marks indicate censored observations. (b) Kaplan–Meier estimates of overall survival by the predefined risk score (Score 1–3), demonstrating stepwise separation across strata (log-rank *p* < 0.0001)

Kaplan–Meier analysis demonstrated significant differences in overall survival across the four chronological age cohorts (*p* = 0.00013; Fig. 1a). Receiver operating characteristic analysis identified a Youden index-derived age cut-off associated with overall survival was statistically significant (*p* < 0.0001; Fig. 1b). The receiver operating characteristic–derived optimal age threshold for overall mortality was 76.5 years (Youden index). Overall survival also differed markedly when stratified by biological age, with significantly impaired survival in patients with ECOG performance status ≥ 2 compared to 0–1 (*p* < 0.0001; Fig. 2a). Finally, combining the receiver operating characteristic-derived age threshold with ECOG performance status (≥ 2) into a simple three-level risk score (1, 2 and 3 score) yielded clear separation of survival curves across score groups (*p* < 0.0001; Fig. 2b).

In multivariable Cox proportional hazards regression adjusting for comorbidity burden and tumor stage, age above the receiver operating characteristic–derived cut-off (> 76.5 years) remained independently associated with worse overall survival (hazard ratio 2.72, 95% confidence interval 1.58–4.68; *p* < 0.001). Higher Charlson Comorbidity Index was also associated with increased mortality (hazard ratio 1.18 per point, 95% confidence interval 1.01–1.38; *p* = 0.040). Pathological tumor stage was strongly associated with overall survival (pT3 vs. ≤pT1: hazard ratio 4.22, 95% confidence interval 1.97–9.02; *p* < 0.001; pT4 vs. ≤pT1: hazard ratio 5.19, 95% confidence interval 2.08–12.94; *p* < 0.001), whereas ECOG performance status ≥ 2, ASA score, nodal status, and sex were not independently associated after adjustment (*Online Resource 2*).

## Discussion

In this contemporary, single-center cohort spanning more than a decade, we observed no statistically significant differences in length of stay or 90-day complication rates across age strata; however, these comparisons may be confounded by temporal evolution in surgical technique and perioperative care. These findings are consistent with the concept that, within a structured perioperative pathway and careful selection, chronological age alone should not be interpreted as a contraindication to radical cystectomy [1, 9]. Importantly, this likely reflects selection of patients deemed suitable for major surgery rather than equivalence in an unselected population.

A key observation is the dissociation between perioperative safety and long-term outcomes. While short-term endpoints were not meaningfully worse with increasing age, overall survival declined across chronological age cohorts and by functional status, consistent with evidence that morbidity and mortality after radical cystectomy remain clinically relevant and are shaped by age, comorbidity, and postoperative events in modern series [1, 10, 11]. Extending this literature, our multivariable Cox model showed that age above the receiver operating characteristic–derived threshold (> 76.5 years) remained independently associated with worse overall survival after adjustment for comorbidity burden and pathological stage, consistent with an age-associated survival gradient that may reflect competing mortality and treatment tolerance in addition to tumor characteristics. This cut-off is a cohort-specific, data-derived threshold intended for pragmatic stratification and should not be interpreted as a biologically universal breakpoint.

Our perioperative findings align with contemporary data suggesting that protocolized perioperative care and centralization can mitigate early morbidity after radical cystectomy [12, 13]. ERAS Society recommendations emphasize standardization and multimodal optimization, which have been associated with shorter length of stay and reduced complications [13]. In ERAS-era care, intensive care unit utilization is typically driven by complications and patient factors rather than routine postoperative management, with higher age and comorbidity linked to unplanned intensive care unit admission and prolonged hospitalization [3, 13]. Randomized evidence further supports comparable oncologic outcomes between robot-assisted and open approaches, with potential perioperative advantages of minimally invasive surgery that may matter in vulnerable patients [6].

These data reinforce that biological age and functional reserve may be more informative than chronological age for risk stratification. Across uro-oncology cohorts, frailty is consistently associated with adverse perioperative endpoints, including intensive care unit–level complications, non-home discharge, and prolonged length of stay [4, 5]. Even simplified frailty tools, such as the 5-item simplified frailty index, can discriminate risk and may outperform traditional surrogates in older subgroups [5]. In our cohort, ECOG performance status provided strong unadjusted survival separation and contributed to a pragmatic bedside score; however, it was not independently associated with overall survival after multivariable adjustment, plausibly due to collinearity with age/comorbidity, limited power, and selection of fitter elderly patients for surgery and underscores that ‘biological age’ in this context reflects a broader selection/fitness construct not fully captured by ECOG alone.

Beyond global functional metrics, objective body-composition markers add prognostic information [14]. Sarcopenia has been associated with worse overall and cancer-specific survival after radical cystectomy independent of comorbidity, supporting muscle depletion as a surrogate of physiological reserve [14]. Together, these data support a multidomain preoperative assessment integrating performance status, frailty, and where feasible objective measures such as sarcopenia to refine counseling and shared decision-making [15, 16].

From an oncologic perspective, the survival decrement with advancing age should be interpreted in the context of competing non-cancer mortality, which is substantial in older strata and cannot be separated using overall survival alone [1, 11, 15]. Because guideline-concordant management of muscle-invasive bladder cancer often requires perioperative systemic therapy and close surveillance, treatment selection should account for functional rather than chronological age [9, 16]. Geriatric assessment offers a structured approach to characterize vulnerabilities across domains (function, comorbidity, cognition, nutrition, social support) and can inform individualized recommendations [16], consistent with concepts that biological aging trajectories, rather than calendar age, shape resilience to oncologic stressors [15]. Accordingly, our combined age-ECOG score should be viewed as complementary to, not a substitute for, formal geriatric assessment where available.

In parallel, bladder-sparing strategies are increasingly considered for selected patients, particularly when frailty or comorbidity raises concern about surgical resilience. Our analysis is restricted to patients selected for RARC and therefore informs counseling within the cystectomy pathway rather than comparative effectiveness versus bladder-preservation approaches. The proposed age–ECOG stratification may nonetheless support shared decision-making when weighing definitive surgery against bladder-sparing strategies in borderline candidates.

Clinically, two implications follow. First, RARC can be offered to carefully selected very old patients without an inevitable penalty in early recovery when embedded in standardized perioperative care pathways [13]. Second, functional status should be incorporated explicitly into preoperative counseling and risk communication, given its linkage to frailty and geriatric vulnerability [5, 16]. Where modifiable deficits are identified, targeted optimization is reasonable; prehabilitation may accelerate functional recovery after radical cystectomy, and ongoing trials may clarify how best to operationalize such programs [17, 18]. The independent association of comorbidity burden with mortality also supports systematic comorbidity assessment and multidisciplinary optimization, particularly in patients above the age threshold.

Limitations include the retrospective, single-center design with inevitable selection effects and potential unmeasured geriatric confounding (e.g., cognition, nutrition, social support), underscoring the value of prospective work incorporating formal geriatric assessment [19, 20]. Given the long accrual period, learning effects, platform refinements, and changes in perioperative pathways could not be disentangled in this retrospective dataset and may have influenced perioperative endpoints. The multivariable Cox analysis was complete-case and showed minor proportional hazards deviations for selected covariates; visual inspection did not suggest clinically meaningful violations and the model was retained. Strengths include a contemporary robotic cohort, inclusion of all diversion types, and standardized 90-day complication reporting using Clavien–Dindo; however, we did not incorporate benchmark-style composite outcomes or detailed complication timing, which may limit clinical granularity [8, 21]. The small ≥ 85-year subgroup limits precision and supports external validation in larger multicenter RARC cohorts.

In summary, age alone should not preclude RARC, as we did not observe statistically significant differences in early recovery or overall complication rates across age cohorts, but these data should not be interpreted as evidence of equivalence, particularly in the smallest (≥ 85 years) subgroup [13]. Long-term outcomes were strongly influenced by host factors; age above a data-driven threshold remained independently associated with overall survival after adjustment, and the combined age-ECOG score may serve as a pragmatic counseling and planning tool pending external validation.

**Table 1:** Baseline patient characteristics stratified by age cohort

Variable	Category / statistic	Cohort 1: <75 years (*n* = 98)	Cohort 2: 75–79 years (*n* = 35)	Cohort 3: 80–84 years (*n* = 32)	Cohort 4: ≥85 years (*n* = 6)	*p* value
Age (years)	Median	65	77	82	86	< 0.001
	Range	34–74	75–79	80–84	85–87	
Sex	Female	5 (5.1%)	1 (2.9%)	4 (12.5%)		0.307
	Male	93 (94.9%)	34 (97.1%)	28 (87.5%)	6 (100.0%)	
ASA score	1	1 (1.0%)				0.084
	2	51 (52.0%)	12 (34.3%)	10 (31.2%)	2 (33.3%)	
	3	44 (44.9%)	23 (65.7%)	22 (68.8%)	3 (50.0%)	
	4	2 (2.0%)			1 (16.7%)	
ECOG performance status	0	38 (38.8%)	2 (5.7%)			< 0.001
	1	41 (41.8%)	19 (54.3%)	13 (40.6%)	4 (66.7%)	
	2	14 (14.3%)	11 (31.4%)	13 (40.6%)	2 (33.3%)	
	3	2 (2.0%)	3 (8.6%)	5 (15.6%)		
	4	3 (3.1%)		1 (3.1%)		

Data are presented as median (range) for continuous variables and n (%) for categorical variables. P values reflect between-cohort comparisons (continuous variables: Kruskal–Wallis test; categorical variables: χ² test or Fisher’s exact test, as appropriate). Abbreviations: ASA, American Society of Anesthesiologists physical status classification; ECOG, Eastern Cooperative Oncology Group performance status.

## Supplementary Information

Below is the link to the electronic supplementary material.

Supplementary Material 1

Supplementary Material 2

## Data Availability

De-identified data supporting the findings of this study are available from the corresponding author upon reasonable request; data sharing is subject to institutional and legal restrictions.

## Code Availability

Statistical code is available from the corresponding author upon reasonable request, subject to institutional policies.

## Declarations

### Use of generative AI and AI-assisted technologies

ChatGPT (GPT-5.2 Thinking; OpenAI) was used solely for grammar and language editing. No new data were generated, and the tool was not used to derive, analyze, or interpret results. The authors reviewed and take full responsibility for the content of the manuscript.
