# Supplementary material for: Chronological and biological age stratify survival after robot-assisted radical cystectomy for bladder cancer: a pragmatic age-ECOG risk score
Source: World J Urol. 2026 Feb 17;44(1):178. doi: 10.1007/s00345-026-06294-4 (PMC12913352; doi:10.1007/s00345-026-06294-4)
Supplement: Supplementary file 1 — Supplementary Material 1 [file 345_2026_6294_MOESM1_ESM.pdf]

Supplementary Tables

**Perioperative complications**	**Category**	**Cohort 1: <75 years (n = 98)**	**Cohort 2: 75–79 years (n = 35)**	**Cohort 3: 80–84 years (n = 32)**	**Cohort 4: ≥85 years (n = 6)**	**p value**
Days operation – discharge	Median	15	17	15	12	0.270
	Range	8–110	9–62	9–24	10–41	
Perioperative complication	Did not occur, n (%)	44 (44.9%)	13 (37.1%)	16 (50.0%)	2 (33.3%)	0.695
	Occurred, n (%)	54 (55.1%)	22 (62.9%)	16 (50.0%)	4 (66.7%)	
CDC	Grade 1, n (%)	9 (16.7%)	1 (4.5%)	5 (31.2%)	1 (25.0%)	0.196
	Grade 2, n (%)	27 (50.0%)	11 (50.0%)	4 (25.0%)	1 (25.0%)	
	Grade 3, n (%)	11 (20.4%)	3 (13.6%)	4 (25.0%)		
	Grade 4, n (%)	6 (11.1%)	3 (13.6%)	1 (6.2%)	1 (25.0%)	
	Grade 5, n (%)	1 (1.9%)	4 (18.2%)	2 (12.5%)	1 (25.0%)	

Supplementary Table 1: Perioperative recovery and postoperative morbidity stratified by age cohort. Data are presented as median (range) for length of stay (operation to discharge) and as n (%) for categorical outcomes. Overall complication occurrence and complication severity according to the Clavien–Dindo classification (CDC) are reported. P values reflect between-cohort comparisons (continuous variables: Kruskal–Wallis test; categorical variables: χ^2 test or Fisher's exact test, as appropriate). Abbreviations: CDC, Clavien–Dindo classification.
