# Supplementary material for: Chronological and biological age stratify survival after robot-assisted radical cystectomy for bladder cancer: a pragmatic age-ECOG risk score
Source: World J Urol. 2026 Feb 17;44(1):178. doi: 10.1007/s00345-026-06294-4 (PMC12913352; doi:10.1007/s00345-026-06294-4)
Supplement: Supplementary file 2 — Supplementary Material 2 [file 345_2026_6294_MOESM2_ESM.pdf]

Variable	HR	95% CI	p-value
Age > 76.5 years (ROC cut-off)	2.72	1.58 – 4.68	<0.001
ECOG ≥2 vs 0–1	0.76	0.41 – 1.41	0.384
ASA (per point)	1.27	0.73 – 2.20	0.392
Charlson Comorbidity Index (per point)	1.18	1.01 – 1.38	0.040
pT2 vs ≤T1	2.76	0.33 – 23.11	0.349
pT3 vs ≤T1	4.22	1.97 – 9.02	<0.001
pT4 vs ≤T1	5.19	2.08 – 12.94	<0.001
N+ vs N0	1.10	0.59 – 2.06	0.757
Female vs Male	1.24	0.44 – 3.51	0.683

Supplementary Table 2: Univariable Cox proportional hazards regression for overall survival after radical cystectomy. Hazard ratios (HRs) are shown with 95% confidence intervals (CIs) and corresponding p values. Age was dichotomized using the receiver operating characteristic–derived cutoff (>76.5 years). ASA was modeled per one-point increase. Pathologic tumor stage was entered as categorical comparisons versus ≤pT1, and nodal status and sex were modeled as binary contrasts. Abbreviations: ASA, American Society of Anesthesiologists physical status classification; CI, confidence interval; ECOG, Eastern Cooperative Oncology Group performance status; HR, hazard ratio; N, lymph node status; pT, pathologic tumor stage; ROC, receiver operating characteristic.
